# Reconstruction of Segmental Mandibular Defects with Double-Barrel Fibula Flap and Osseo-Integrated Implants: A Systematic Review

**DOI:** 10.3390/jcm13123547

**Published:** 2024-06-17

**Authors:** Saad Khayat, Ángela Sada Urmeneta, Borja González Moure, Diego Fernández Acosta, Marta Benito Anguita, Ana López López, Juan José Verdaguer Martín, Ignacio Navarro Cuéllar, Farzin Falahat, Carlos Navarro Cuéllar

**Affiliations:** 1 Maxillofacial Surgery Department, Hospital Gregorio Marañón, 28007 Madrid, Spain; saad.khayat@gmail.com (S.K.); angela.sada15@gmail.com (Á.S.U.); borjagonzalezuam@gmail.com (B.G.M.); diegofernandezacosta77@gmail.com (D.F.A.); martabenito0910@hotmail.com (M.B.A.); anitalopez@gmail.com (A.L.L.); juanjoverdaguer@hotmail.com (J.J.V.M.); nnavcu@hotmail.com (I.N.C.); 2 Surgery Department, School of Medicine, Universidad Complutense de Madrid, 28040 Madrid, Spain; 3 Maxillofacial Surgery Department, Hospital Clínico San Carlos, 28040 Madrid, Spain

**Keywords:** dental implants, double-barrel, fibula flap, mandibular reconstruction

## Abstract

**Background:** Mandibular defects resulting from oncological treatment pose significant aesthetic and functional challenges due to the involvement of bone and soft tissues. Immediate reconstruction is crucial to address complications such as malocclusion, mandibular deviation, temporomandibular joint (TMJ) changes, and soft tissue retraction. These issues can lead to functional impairments, including difficulties in chewing, swallowing, and speech. The fibula flap is widely used for mandibular reconstruction due to its long bone segment and robust vascular supply, though it may not always provide adequate bone height for optimal dental rehabilitation. This systematic review aims to determine if the double-barreled fibula flap (DBFF) configuration is a viable alternative for mandibular reconstruction and to evaluate the outcomes of dental implants placed in this type of flap. **Materials and Methods:** This study adhered to the Cochrane Collaboration criteria and PRISMA guidelines and was registered on the International Platform of Registered Systematic Review and Meta-Analysis Protocols Database (INPLASY2023120026). We included clinical studies published in English, Spanish, or French that focused on adult patients undergoing segmental mandibulectomy followed by DBFF reconstruction and dental rehabilitation. Data sources included Medline/PubMed, the Cochrane Library, EMBASE, Scopus, and manual searches. Two reviewers independently screened and selected studies, with discrepancies resolved by a third reviewer. Data extraction captured variables such as publication year, patient demographics, number of implants, follow-up duration, flap survival, implant failure, and aesthetic outcomes. The risk of bias was assessed using the JBI appraisal tool, and the certainty of evidence was evaluated using the GRADE approach. **Results:** A total of 17 clinical studies were included, evaluating 245 patients and 402 dental implants. The average patient age was 43.7 years, with a mean follow-up period of 34.3 months. Flap survival was high, with a 98.3% success rate and only four flap losses. The implant failure rate was low at 1.74%. Esthetic outcomes were varied, with only three studies using standardized protocols for evaluation. The overall certainty of evidence for flap survival was moderate, low for implant failure, and very low for aesthetics due to the subjective nature of assessments and variability in reporting. **Conclusions:** The primary limitations of the evidence included in this review are the observational design of the studies, leading to an inherent risk of bias, inconsistency in reporting methods, and imprecision in outcome measures. Additionally, the subjective nature of aesthetic evaluations and the variability in assessment tools further limit the reliability of the findings. The DBFF technique demonstrates excellent outcomes for mandibular reconstruction, with high flap survival and low implant failure rates, making it a viable option for dental rehabilitation. However, the evidence for aesthetic outcomes is less certain, highlighting the need for more rigorous and standardized research. This review supports the DBFF as a good alternative for mandibular reconstruction with successful dental implant integration, although further studies are needed to enhance the reliability of aesthetic evaluations.

## 1. Introduction

In patients undergoing oncologic treatment, mandibular defects raise significant challenges affecting both bone and soft tissues, resulting in significant aesthetic and functional disorders. In these cases, the immediate reconstruction of the mandible is imperative. Segmental defects in the mandible can lead to complications such as malocclusion, mandibular deviation [1,2], temporomandibular joint (TMJ) [2] disorders, and soft tissue retraction [2,3]. Removal of the symphysis and mandibular body during segmental mandibulectomy can cause retrusion of the lower third of the face, along with lower lip ptosis and lip incompetence. Facial asymmetry is evident in cases where the resection involves the mandibular body, resulting in soft tissue collapse on the affected side. In addition to lip incompetence, patients may experience salivary incontinence and difficulties with chewing, swallowing, and speech articulation [2,4,5]. Those who undergo mandibular resection without reconstruction face progressive deviation toward the affected side and retrusion, which increases functional problems. In addition, the replacement of vertical chewing movements with oblique and diagonal movements, controlled by a single temporomandibular joint, together with limited tongue mobility in many cases, further complicates the social interactions of these patients [6,7].

In addressing segmental defects in the mandible and adjacent soft tissues, free flaps are widely acknowledged as the primary reconstructive method [8,9]. Different flaps providing bone and soft tissues have been described, such as the fibula flap, scapular flap, and iliac crest flap [9,10,11,12]. The fibula flap has become more widely used in recent decades as it offers a long bone segment suitable for reconstructing defects over 10 cm in length [13,14]. It also has a strong medullary and periosteal vascular supply, a long and reliable vascular pedicle, a thin, pliable and substantial skin paddle for soft tissue reconstruction, and bicortical bone ideal for dental rehabilitation using osseo-integrated dental implants.

However, despite its advantages, the fibula flap may be insufficient for providing adequate bone height [15]. The vertical mismatch between the remnant mandible and the fibula flap represents a challenge for dental rehabilitation, potentially causing long-term issues with implant overloading and compromising both functional and aesthetic outcomes. Some options have been described to solve this problem [15,16,17,18,19,20], such as positioning the fibula flap superiorly, causing a basal defect; guided bone regeneration, deferring the implant rehabilitation; or a double-barrel fibula flap [21,22], with the risk of compromising the vascular supply to the more distal segment.

A systematic review was conducted on double-barrel fibula flaps and dental implant rehabilitation for segmental mandibular reconstruction, with the aim of evaluating the functional results, the survival of the flap, the placement of dental implants, and osseointegration rate.

## 2. Materials and Methods

This study followed the criteria outlined by the Cochrane Collaboration [23] and adhered to the guidelines set by the Preferred Reporting Items for Systematic Reviews and Meta-Analysis (PRISMA) statement [24]. Registration was completed on the International Platform of Registered Systematic Review and Meta-Analysis Protocols Database (INPLASY) under the reference INPLASY2023120026. The analysis centered on the population, intervention, control, outcome (PICO) index.

### 2.1. Eligibility Criteria

This systematic review included studies that met the following criteria:

- Language: Studies were eligible if they were published in English, Spanish, or French. This language restriction was applied to ensure that the reviewers could accurately interpret the study findings.
- Availability: Only studies with the full text available were included. Abstracts, conference proceedings, and other forms of partial data were excluded.
- Participants: The included studies focused on adult patients who underwent segmental mandibulectomy followed by reconstruction using a double-barreled fibula flap and subsequent dental rehabilitation.
- Study Type: Clinical studies were considered for inclusion. In vitro studies, animal studies, and studies with non-human subjects were excluded to maintain relevance to clinical practice.
- Data Completeness: Studies with incomplete data or non-retrievable data were excluded. Only studies with sufficiently detailed and complete data to allow for comprehensive analysis were included.

### 2.2. Information Sources

The search for relevant studies involved a comprehensive exploration of several electronic databases. The databases searched included:

- Medline/PubMed.
- Cochrane Library.
- EMBASE.
- Scopus.

The search strategy was designed to be exhaustive and inclusive of all potentially relevant studies up to August 2023. Additionally, manual searches were performed using PubMed and Google Scholar to identify any additional articles that might have been missed in the initial database search. This manual search ensured that the review captured the most recent and relevant studies.

### 2.3. Search Strategy

The search strategies were customized for each database to maximize the retrieval of relevant studies. In PubMed, the following search terms and Boolean operators were used:

’ ”fibula/transplantation”[MeSH Terms] OR “fibula/transplantation”[Title/Abstract] OR (“free tissue flaps”[Title/Abstract] OR “free tissue flaps”[MeSH Terms]) AND (“Dental Implants”[MeSH Terms] OR “dental implant*”[Title/Abstract]) AND (“Mandible/surgery”[Title/Abstract] OR “mandible/surgery”[MeSH Terms]). In Scopus, the search terms included: ‘ALL (mandibulectomy) AND ALL (fibula AND flap) AND TITLE-ABS-KEY (dental AND implant)’. For the Cochrane Library, the search query was: ’mandible AND surgery in Title Abstract Keyword AND fibula in Title Abstract Keyword AND dental implant in Title Abstract Keyword‘. The search query for EMBASE followed the population, intervention, control, outcome (PICO) format and is detailed in Appendix A.

The selection of articles involved a multi-step process using the Rayyan AI platform to streamline the screening and selection phases. Initially, two independent reviewers (SK and DFA) screened the titles and abstracts of the identified studies. If there were any discrepancies between the reviewers regarding the eligibility of an article, a third reviewer (BGM) resolved these conflicts. Articles that were deemed eligible based on their titles and abstracts were then retrieved in full text for a more detailed assessment.

The full-text articles were thoroughly analyzed by the same two reviewers (SK and DFA). Any further disagreements were again resolved by the third reviewer (BGM). This process ensured that the selection of studies was objective and unbiased.

Data extraction was carried out using a standardized form created in Microsoft Office Excel (version 2405). The data extraction form was designed to capture all relevant variables, including:

- Year of publication.
- Country of origin.
- Type of study.
- Number of patients.
- Age and gender of patients.
- Number of implants.
- Follow-up duration.
- Main cause for initial surgery.
- Adjuvant radiation therapy.
- Flap survival.
- Implant failure.
- Mandibular defect.

Each study was reviewed in detail to extract this data. The data extraction process was overseen by all reviewers to ensure consistency and accuracy. Any discrepancies in data extraction were discussed and resolved through consensus among the reviewers.

### 2.4. Data Items

Outcomes Sought:

We sought data on the following primary and secondary outcomes from each study included in this review.

Primary Outcomes:

Flap Survival: Defined as the successful integration and function of the double-barreled fibula flap without necrosis or significant complications. The mandibular defect in centimeters was calculated whenever it was stated in the studies. The average defect size was calculated for each article where this information was provided.

Implant Failure Rates: Defined as the number of dental implants that failed to osseo-integrate or were lost during the follow-up period. Only explicitly stated implant failures or survival were included; if no implant loss was reported, it was assumed there were no losses. Only explicitly stated implant failures or survivals were included in our analysis. Follow-up duration was included as reported. If not explicitly stated, it was not included in the follow-up duration analysis. For studies that reported a follow-up duration, we calculated both the average and median follow-up periods.

Secondary Outcomes:

Functional Outcomes: Including masticatory function, speech function, and aesthetic outcomes.

Adverse Events: Including any reported complications related to the surgical procedure or the dental implants.

For each outcome domain, all results compatible with the domain, including various measures, time points, and analyses provided by the studies, were collected. Multiple measures or time points were extracted where available.

### 2.5. Risk of Bias in Individual Studies

The risk of bias in the included studies was assessed using the JBI appraisal tool. The JBI tool was selected for its comprehensive criteria tailored to different types of study designs. The specific checklists used from the JBI tool in this review were the Randomized Controlled Trials (RCTs) Checklist, the Cohort Studies Checklist, and the Case Series Checklist. The JBI appraisal tool evaluates various aspects of study design and execution to determine the potential for bias. Two reviewers (SK and DFA) independently assessed each study using the JBI appraisal tool. They evaluated each domain and provided judgments categorized as “low risk”, “high risk”, or “unclear risk” of bias. Any discrepancies between the two reviewers were discussed and resolved through consensus. If consensus could not be reached, a third reviewer (BGM) was consulted to make the final decision. The overall risk of bias across the included studies was summarized and presented in a risk of bias table.

### 2.6. Summary Measures

The principal summary measures used in this review included flap survival rates and implant failure rates. The proportion (or percentage) of successful flap integrations without necrosis or significant complications is reported as a simple proportion with a 95% confidence interval (CI) to provide an estimate of the survival rate within the group. The percentage of failed implants during the follow-up period was calculated as the number of implant failures divided by the total number of implants. The incidence of complications related to the surgical procedure or dental implants was also calculated. For each outcome, we reported descriptive statistics, including means, medians, ranges, and proportions where applicable.

### 2.7. Synthesis of Results

Given the heterogeneity of the included studies, a qualitative synthesis of the results was conducted. The variability in study design, patient populations, and outcome measures precluded the possibility of a quantitative meta-analysis. Therefore, the findings are presented in a narrative format, summarizing the key outcomes and trends observed across the studies.

We tabulated the characteristics of each study, including intervention type, patient demographics, study design, and outcomes measured. Studies that met the eligibility criteria and had relevant data for the outcomes of interest were included in the synthesis. To ensure consistency across studies, data were converted to a common format. For example, follow-up durations reported in different units (e.g., months, years) were standardized to months. Additionally, where necessary, effect sizes were calculated or converted to a common metric to facilitate comparison. The results from individual studies were systematically tabulated to summarize key findings, including outcome measures and effect sizes. Given the heterogeneity in study designs, populations, and outcomes, a narrative synthesis was performed. This involved summarizing the key findings of each study and providing context for the interpretation of the data. The rationale for choosing narrative synthesis over meta-analysis was based on the diversity of the included studies, which precluded meaningful quantitative synthesis. Although not performed in this review, if meta-analysis had been feasible, we would have used a random effects model to account for between-study heterogeneity. We would have assessed heterogeneity using the I² statistic and conducted sensitivity analyses to explore the impact of study characteristics on the pooled estimates.

If appropriate data were available, meta-regression would have been used to investigate the relationship between study-level characteristics (e.g., study design, sample size) and the effect sizes. However, due to the limited number of studies and their heterogeneity, meta-regression was not conducted.

In the absence of a meta-analysis, the risk of reporting bias was assessed by examining the methods sections of the included studies to identify any evidence of selective reporting of outcomes. Studies were evaluated for discrepancies between pre-specified outcomes and reported outcomes. A comprehensive search strategy was employed across multiple databases and supplemented by manual searches to minimize the risk of missing relevant studies.

### 2.8. Certainty Assessment

To assess the certainty (or confidence) in the body of evidence for each outcome, the Grading of Recommendations Assessment, Development and Evaluation (GRADE) approach was used. The certainty of evidence was evaluated across five domains: risk of bias, consistency of effect, imprecision, indirectness, and publication bias. The variables chosen for the certainty assessment were based on the clinical importance and the main subject of this review: flap survival, implant failure, and cosmetics. GRADE summary tables were created to present the overall certainty of the evidence for each key outcome, providing a clear and transparent assessment.

## 3. Results

### 3.1. Study Selection

**Figure 1:**
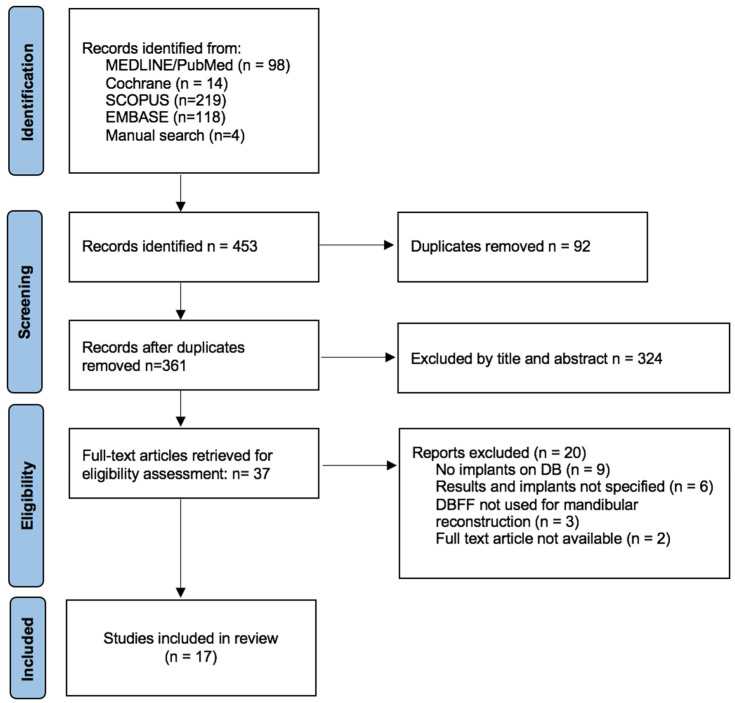
Flowchart for the study selection.

The flowchart for the study selection process is shown in Figure 1. The initial search resulted in 453 titles identified: 98 studies from MEDLINE/PubMed, 118 from Embase, 14 from Cochrane, 219 from SCOPUS, and a further manual search of references of relevant articles revealed 4 more articles. A total of 92 duplicates were found and removed, which yielded a total of 361 studies. Based on titles and abstracts, population and study type, 324 titles were excluded. Out of the 37 studies selected for full-text analysis, 20 were excluded after further reading, owing to the following reasons:

- Implants not being placed on the double-barrel fibula flap (9);
- Results were not shown specifically for the mandibles and implants placed on the DBFF (6);
- DBFF not used for mandibular reconstruction (3);
- Full text not available (2).

Finally, a total of 17 clinical studies were included in this review. The Kappa score between reviewers was 0.94.

### 3.2. Study Characteristics

The study characteristics are displayed in Table 1.

The publication dates ranged from 2006 to 2022. A total of 245 patients and flaps were evaluated, and a total of 402 dental implants were placed on the double-barrel fibula flaps. The patients had a mean age of 43.7 years old, 43.4% were female, and 56.6% were male (in three cases gender was not specified).

The follow-up period ranged from 12 months to 60 months, with an average follow-up time of 34.3 months.

### 3.3. Risk of Bias

Because of the heterogeneity among the type of studies, it was necessary to study the risk of bias using methodological quality assessment tools. The Joanna Briggs Institute (JBI) [41] critical appraisal checklist was utilized for each study type to evaluate the risk of bias across multiple domains (Table 2).

#### 3.3.1. Cohort Studies

All cohort studies were consistent in recruiting participants from similar populations, ensuring comparability at baseline. The exposure was measured similarly across all studies. The identification of confounding factors was less consistent. Several studies did not adequately identify or report confounding factors (noted as “U” for unclear or “N” for no), which could affect the validity of the results by introducing potential biases. None of the studies stated strategies for handling confounding factors, indicating a gap in addressing potential biases that could influence the outcomes. All participants were free of the outcome at the start of the study, which helps to reduce the risk of reverse causality and supports the temporal relationship between exposure and outcome. Outcomes were measured in a valid and reliable way across all studies. The follow-up period was reported and deemed sufficient in all studies. Most studies did not utilize strategies to address incomplete follow-up. All studies used appropriate statistical analysis methods, which strengthened the validity of the conclusions drawn from the data.

#### 3.3.2. Case Series

All case series had clear inclusion criteria, ensuring that the participants included were relevant to the study objectives. The condition of interest was measured consistently across all participants. Valid methods were used to identify the condition in all case series. The inclusion of participants was not always consecutive, with some studies marked as “U” or “N”, potentially introducing selection bias. Most case series had complete inclusion of participants, but some did not, which could affect the representativeness of the findings. Demographic information was generally well-reported, though some studies did not provide clear details, potentially limiting the generalizability of the results. Clinical information was not always clearly reported, with some studies marked as “N” or “U”, which could affect the interpretability of the findings. Outcomes were clearly reported in all studies. Reporting on the presenting site demographics was inconsistent. Most case series used appropriate statistical analysis methods.

#### 3.3.3. Case Reports

Demographic characteristics were clearly described in all case reports, providing context for the reported cases. The patient’s history was not always clearly described, with some studies failing to present this information as a timeline, potentially affecting the comprehensiveness of the case report. The current clinical condition was clearly described in all reports, ensuring that the reader understood the patient’s status at the time of presentation. Diagnostic tests and assessment methods were clearly described in all reports, supporting the validity of the diagnosis and subsequent treatment. The interventions or treatment procedures were clearly described. The post-intervention clinical condition was clearly described, allowing for an assessment of the treatment outcomes. Adverse events were not consistently identified or described, which could limit the understanding of potential risks associated with the interventions. All case reports provided takeaway lessons, summarizing the key insights and implications for clinical practice.

The studies generally exhibited strong methodologies in measuring exposures and outcomes validly and reliably. Appropriate statistical analyses were consistently used across studies.

Most studies had clear inclusion criteria and complete reporting of key demographic and clinical information. The identification and handling of confounding factors were often inadequate, which could introduce bias and affect the validity of the findings. Follow-up completeness and strategies to address incomplete follow-up were not consistently reported, potentially leading to attrition bias. Some case series and case reports lacked detailed reporting of patient history and adverse events, which could limit the comprehensiveness and applicability of the findings.

### 3.4. Results of Individual Studies

The results are displayed in Table 3. This table provides the characteristics and key outcomes of the included studies. It includes the number and percentage of patients with malignancies, the use of radiation therapy, flap survival rates, implant failure rates, and the average mandibular defect size. The studies are listed by author, with the number of malignancies and radiation therapy sessions reported. Flap survival is shown as the number of successful integrations versus total flaps, and implant failures are noted where applicable. The average mandibular defect size is provided in millimeters for each study. Overall, the table highlights a flap survival rate of 98.3% and an implant failure rate of 1.74%, with the average mandibular defect size being 75.6 mm.

**Figure 2:**
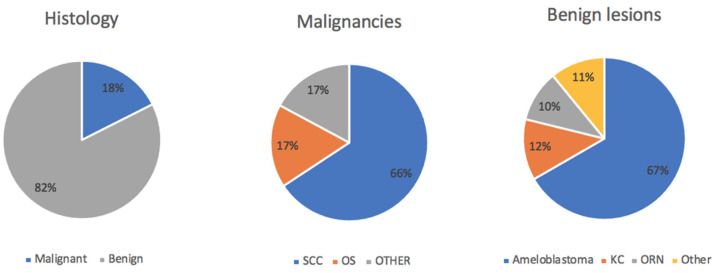
SCC: squamous cell carcinoma; OS: osteosarcoma; KC: keratocyst; ORN: osteoradionecrosis.

Regarding the etiology, 43 out of 245 patients were operated on due to malignancy (17.6%), with benign lesions comprising the majority of cases where DBFF was performed. Among the malignant etiologies, 88.4% (38) were squamous cell carcinoma, 7% (3) were osteosarcoma, and 4.6% (2) were other types of malignancies (Figure 2).

Among the benign lesions (168), the first cause was ameloblastoma, with 104 patients diagnosed (61.9%); followed by keratocysts (11.3%, 19 patients); and finally osteoradionecrosis (9.5%, 16 patients) (Figure 1). Other benign pathologies and traumas made up the rest of the cases. A total of 10% of patients underwent adjuvant radiotherapy.

The length of the defect was only measured by selected authors, namely, Antúnez-Conde et al. [25], Bianchi et al. [27], Chang et al. [30], Cuéllar et al. [15], He et al. [32], Margabandu et al. [34], and Ruhin et al. [37]). The defect length ranged from 40 mm (Bianchi et al. [27]) to 106 mm (Antunez-Conde et al. [25]), with an average defect length of 75.61 mm.

A total of four articles mentioned the fibula flap length harvested (Chang et al. [28], Cuellar et al. [15], Margabandu et al. [34], and Shen et al. [38]), with its average being 175.6 mm. There were six authors who examined the vertical height achieved by double-barreling the flap, with results ranging from 11.8 mm up to 34.2 mm (Antunez-Conde [25], Chang et al. [29], Cuellar et al. [15], He et al. [32], Margabandu et al. [34], and Wang et al. [40]).

Flap survival was rated at 98.3% with a total flap loss of 4. In total, 23 flap complications were identified [15,29,34,36,38,39] including infection, hardware failure, hardware exposure, bone exposure, and vascular thrombosis.

Bone resorption was identified in five studies (Antunez-Conde [25], Chang et al. [29], Chen et al. [31], Cuellar et al. [15], and Wang et al. [40]). It ranged from 0.42 mm to 1.23 mm. Chen et al. [31] and Wang et al. [40] followed the resorption during 3 years, reporting 0.75 mm and 0.68 mm, at the 3 year follow-up.

Of the 423 implants placed, only 7 were lost during the follow-up period (1.65%). Not every author specified the moment for placing the dental implant, such as (Chang et al. [29], Chen et al. [31], He et al. [32], and Qu et al. [36]). Cuellar et al. [15], Margabandu [34], Paranque [35], and Ruhin [37] opted for delayed implants. A total of nine studies [25,26,27,28,30,33,38,39,40] performed an immediate implant placement. Shen et al. [38] and Wang et al. [40] opted for both options, immediate and deferred rehabilitation, the former in three patients and the latter in one patient, respectively. A total of 78 implants were placed immediately. Only one article (Wang et al. [40]) addressed the primary stability of the implants placed in a DBFF, achieving good results, with an average implant stability quotient (ISQ) of 78 ± 7.1.

In the area of cosmetic results, only three studies directly addressed this aspect. In a total of 76 patients studied, good to excellent aesthetic results were achieved describing a standardized protocol for measuring the aesthetic results in a quantitative and score-based fashion (Chen et al. [31], Margabandu et al. [34], and Shen et al. [38]). Antunez-Conde [25], Berrone [26], Chang [28], Li [33], Qu [36], and Ruhin [37] mentioned the aesthetic results in a qualitative manner, achieving a satisfactory facial contour and good aesthetic results. Bianchi [27], Chang [29], Chang [30], Cuellar [15], Paranque [35], and Trilles [39] only mentioned the aesthetic results of their patients in their discussion and/or conclusion. He [32] and Wang [40] did not mention the aesthetics in their respective articles.

### 3.5. Quality of Evidence

The quality of evidence based on the GRADE system was assessed as high-, moderate-, low-, and very low-quality evidence. Since all the studies included were observational in nature, the level of evidence was downgraded by one point. The main factors leading to downgrading were indirectness, imprecision, and inconsistency (Table 4).

The significant heterogeneity found in this systematic review is based on the rarity of this double-barrel technique, with the majority of articles being case reports or series of cases. Additionally, the significant difference among population types, many focused on non-oncological causes, influences the adjuvant treatments such as radiotherapy, which substantially increases the complication rate and implant loss. The lack of homogeneity in collecting variables further complicates the data collection process.

## 4. Discussion

Mandibular reconstruction with a fibula flap after segmental mandibulectomy is the most common treatment for a complete restoration of all necessary tissues to achieve good function and aesthetics, including dental rehabilitation [14].

The fibula diaphysis is straight and has a triangular shape, which can create a mismatch issue when it is attached to a higher or dentate mandible. The height discrepancy between the remnant mandible and the fibula flap may lead to problems for the functional rehabilitation of patients as it generates an unfavorable implant/crown ratio. Therefore, patients often require additional surgery to increase the height of the bone [15]. Using bone grafts is a common and dependable solution for this purpose [15,25]. Some surgeons also use distraction techniques to restore the bone height [15,20,29,40]. However, because of its limited space and technical difficulty, it is not generally used as a first line treatment for vertical discrepancy.

The double-barrel conformation [21,22] of the fibula flap solves the vertical discrepancy described in other studies that use the single fibula flap, hindering the implantological rehabilitation of these patients. The mandibular height restored with the double-barrel configuration ensures a good facial contour and an acceptable alveolar ridge for implant placement [31]. Thus, the objective with this fibula flap modification should be an optimal dental prosthetic rehabilitation and the restoration of the premorbid state of the patient in terms of aesthetics and function.

Adequate surgical technique performed by an experienced team is one of the main conditioning factors for the survival of the flap. Adequate flap harvest and microsurgical techniques are imperative to guarantee adequate blood supply, and therefore ensuring flap viability. This is imperative to facilitate implant osseointegration. However, using the DBFF technique may pose challenges [38,39] to vascularization due to disruptions in periosteal blood supply caused by segmentation, and the distance of the distal fibula segment from the main pedicle. Additionally, the twisting or compression of blood vessels can result in venous thrombosis [29,34,38], leading to ischemia and potential loss of the flap. It seems reasonable to only consider this type of configuration in very specific cases. The most suitable clinical cases are those involving losses of substance affecting the dentate portion of the mandible, including a portion of its horizontal branch. These are patients in which it is desirable to restore mandibular height to maintain the level of the alveolar crest and allow proper alignment of future implant-supported prosthetic rehabilitation with the adjacent dental level. Bone defects up to 15 cm remain suitable for this double-barrel configuration [37]. As it is generally considered that the length of the fibula flap can reach 22 cm, it is reasonable to preserve at least 3 cm of the distal segment to conform the upper part of the reconstruction, and in osteotomies, an additional 2 cm can be considered. Some authors modified the technique by performing a vertical osteotomy and subperiosteal reflection without excising the bone segment at the bending region, which can decrease the likelihood of damaging the vascular pedicle while preserving bone stock. A few publications address the number of osteotomies performed, but there seems to be a tendency toward four segments with three osteotomies, although He’s [32] osteotomies allow for up to six segments. Other advantages of using the double-barrel flap are the following: no deferred surgery in contrast to the onlay bone graft and fibula distraction for implants placement, availability of a skin paddle in cases where soft tissue reconstruction may be required (especially in malignant tumors) or as a monitoring device for flap viability, and better biomechanical loads on the implants and on the underlying bone.

However, it is imperative to mention that the number of patients with malignant tumors requiring subsequent radiotherapy is low, which decreases the likelihood of flap complications such as radionecrosis and implant failure. In addition, the mean age of the patients included in the study is also low, making them a population with fewer comorbidities and peripheral circulation disorders, which also enhances flap survival. Therefore, it would be pertinent for future investigations to analyze the incidence of complications within a cohort similar to the demographic profile under examination in this study, compared with a group consisting of elderly individuals. Additional investigations encompassing a wider age spectrum or specifically targeting the elderly population are warranted to elucidate potential age-related impacts on both flap survival and dental implant outcomes. Benign lesions (benign tumors, cysts, trauma, ORN) accounted for the most frequent cause for segmental mandibulectomy and subsequent reconstruction with a double-barrel configuration of the fibula flap. The lack of complementary treatment in these type of patients (no radiation therapy, and generally, no chemotherapy) also allow for a safer and more straightforward approach when planning surgery and immediate implant placement. Surgical time is also shorter, because only the mandible has to be treated, with no neck dissection or intraoperative radiotherapy for the patient.

The use of dental implants for oral rehabilitation in fibula flaps has been widely used after mandibular reconstruction and has demonstrated consistent effectiveness (Taylor et al., 1989 [8]; Hidalgo, 1989 [9]). Chang et al., [42] highlighted that placing dental implants in fibula flaps concurrently during microvascular free tissue transfer provides enhanced adaptability for reconstructing a precise interarch relationship using a streamlined method. Nevertheless, immediate implant placement could potentially compromise bone viability, extend the surgical procedure, or lead to implant misalignment. Another interesting aspect of the double-barrel fibula flap is the theoretical increased rate of simultaneous implant placement during reconstructive surgery, as mentioned by Trilles [39]. Immediate dental implants offer the advantage of promptly restoring mastication and speech, potentially slowing down fibula resorption to some degree, and decreasing the frequency and cost of surgeries. Immediate implant placement is typically recommended for patients with non-neoplastic conditions, benign tumors, or malignancies that do not require postoperative radiotherapy, with the added benefit of immediate loading if the primary stability is acceptable, either by periotest measurement, moment of force or torque, or by resonance frequency analysis (RFA) [43]. Implant stability quotient (ISQ) values, measured by RFA, greater than 65 have been regarded as most favorable for implant stability, as was the case in the study of Wang et al. [40], where the average ISQ was 78. On the other hand, delayed implant placement is recommended for patients undergoing the excision of other malignant tumors. The success of immediate dental implants depends on other factors [44]: primary insertion may impact the blood supply to the fibula flap; postoperative radiation therapy could increase the risk of implant failure; and dental implants may interfere with bone regeneration capacity, extending the bone healing process. Also, the placement of simultaneous dental implants has been reported to notably increase the complexity of presurgical planning, as the surgical team must take into account not only the possible interference with the plate and screws, but also the final occlusion desired with the dental prostheses.

The evidence for aesthetic outcomes, encompassing both cosmetic and functional aspects, is of very low certainty. This is attributed to several factors, including serious risk of bias due to the subjective nature of the assessments, inconsistency in reporting methods, very serious indirectness as not all studies directly addressed aesthetics using standardized measures, and imprecision stemming from variability in the results. Only a few studies employed standardized protocols for evaluating aesthetic outcomes, while others relied on qualitative or discussion-based mentions of satisfactory results. Consequently, our confidence in the aesthetic findings is minimal, highlighting the need for more rigorous and standardized research in this area.

In addition to its significant aesthetic advantages concerning mandibular height, the double-barrel configuration offers distinctive biomechanical benefits that could enhance its suitability for dental implant placement compared to a single configuration. The single-barrel design typically positions the fibula near the lower border of the mandible, requiring the prosthetic dental implant to extend further to achieve occlusion, leading to an unfavorable crown/implant ratio and increase occlusal loading that may compromise implant survival [45]. In contrast, the double-barrel design elevates the implant-containing layer to the level of the alveolar ridge, reducing the distance needed for occlusion and minimizing implant susceptibility to overloading. This positioning allows dental implants to be placed higher in the mandible, decreasing the risk of implant overload and resulting in a lower crown-to-implant ratio.

Another critical factor for implant survival and prosthesis stability are the soft tissues surrounding the dental implants and trying to avoid peri-implantitis and bone resorption. The fibula flap offers the benefit of supplying sufficient length and high-quality bone for reconstruction purposes; however, it frequently substitutes the mucosa with a thick skin paddle, with a completely different behavior to that of the oral mucosa. This paddle can be eventually undermined if implants are required. However, Chang et al. [29] reported that patients with the combination of palatal mucosal graft and a double-barrel fibula flap showed better results in terms of implant survival and bone resorption.

In this review, the low rate of implant failure during follow-up (1.65%, 7 out of 423), makes the double-barrel fibula flap a good alternative for dental rehabilitation and facial contour restoration. This is in agreement with what was observed in the study by Faverani LP et al. [46], with similar rates of flap and implant loss. While the studies generally employed strong methodologies, the indirectness introduced by the omission of implant failure data in two studies, along with the potential for publication bias, necessitates caution in interpreting these results. Although the observed implant failure rates are consistent and precise, the overall confidence in the effect estimate remains limited.

Overall, while the included studies demonstrate several methodological strengths, there are notable areas where improvements are needed to reduce potential biases and enhance the reliability of the evidence. Furthermore, while the evidence for flap survival is moderately certain and suggests a reliable outcome, the findings for implant failure and aesthetics are less certain and should be interpreted with caution. These variations underscore the importance of addressing methodological limitations and standardizing outcome measures in future studies to enhance the reliability and applicability of the evidence in reconstructive surgery using double-barreled fibula flaps. Addressing these limitations in future research will be crucial for strengthening the body of evidence in this field.

Future studies are needed to reduce implant-supported prosthetic rehabilitation time when performing mandibular reconstruction with double-barrel fibula flaps and implants. Through virtual planning and guided surgery using CAD/CAM techniques, the virtual planning of immediate implants without interference with the osteosynthesis material is possible. However, studies must be performed to allow the immediate prosthetic loading of implants placed in the double fibula flap.

## 5. Conclusions

In view of the results, we can state that the double fibula flap (DBFF) shows promising results for the reconstruction of mandibular defects, with a high flap survival rate and a low implant failure rate. However, the evidence should be interpreted with caution due to the moderate certainty of flap survival, the low certainty of implant failure, and the very low certainty of aesthetic results as assessed by the GRADE system. The high flap survival rate (98.3%) and low implant failure rate (1.74%) suggest that DBFF is a viable option for mandibular reconstruction and dental rehabilitation. However, further rigorous and standardized research is needed to improve the reliability of these results and to fully determine the efficacy and safety of the DBFF technique in several different patient populations.

## Figures and Tables

**Table 1:** Demographic and Study Characteristics of Included Studies.

AUTHORS	YEAR	COUNTRY	TYPE OF STUDY	NUMBER OF	AGE (AVERAGE)	GENDER	Nº	FOLLOW UP
				PATIENTS			IMPLANTS	
Antúnez-Conde et al. [25]	2021	Spain	Retrospective	5	46.4	4M 1F	20	26 months
Berrone et al. [26]	2020	Italy	Case reports	1	52	1F	2	*
Bianchi et al. [27]	2013	Italy	Retrospective	9	53.8	4M 5F	32	53.6 months
Chang et al. (1) [28]	2006	Taiwan	Case reports	1	46	1F	2	12 months
Chang et al. (2) [29]	2014	Taiwan	Retrospective	13	41.8	9M 4F	36	55.8 months
Chang et al. (3) [30]	2011	Taiwan	Retrospective	10	41.1	7M 3F	25	22 months
Chen et al. [31]	2018	China	Retrospective	12	34.7	6M 6F	33	36 months
Cuéllar et al. [15]	2021	Spain	Retrospective	8	56.6	5M 3F	28	*
He et al. [32]	2011	China	Case reports	7	40	4M 3F	*	28 months
Li et al. [33]	2023	China	Case reports	1	47	1F	5	*
Margabandu et al. [34]	2023	India	Retrospective	26	42.1	16M 10F	54	16.5 months
Paranque et al. [35]	2011	France	Case reports	1	49	1M	6	60 months
Qu et al. [36]	2017	China	Retrospective	52	41.4	24M 28F	95	*
Ruhin et al. [37]	2006	France	Case reports	3	37		11	*
Shen et al. [38]	2012	China	Retrospective	45	36.4	24M 21F	*	34.7 months
Trilles et al. [39]	2022	Us	Retrospective	42	34.2	27M 15F	29	24 months
Wang et al. [40]	2013	China	Retrospective	9	41.1	6M 3F	24	42.5 months
				**245**	**43.56**	**137M 105F**	**402**	

This table provides detailed demographic and study characteristics of the included studies. It lists each study by author, year of publication, country, type of study, number of patients, average age, gender distribution, number of implants placed, and follow-up duration. The total number of patients across all studies is 245, with an average age of 43.56 years. Gender distribution includes 137 males and 105 females. A total of 402 implants were placed across the studies. * Not specified in the articles.

**Table 2:** Risk of bias assessment for included studies Using the Joanna Briggs Institute (JBI) appraisal tool [41].

**COHORTS**	**Antunez-Conde [25]**	**Bianchi [27]**	**Chang [29]**	**Chen [31]**	**Cuellar [16]**	**Trilles [39]**	**Wang [40]**
Were the two groups similar and recruited from the same population?	Y	Y	Y	Y	Y	Y	Y
Were the exposures measured similarly to assign people to both exposed and unexposed groups?	Y	Y	Y	Y	Y	Y	Y
Was the exposure measured in a valid and reliable way?	Y	Y	Y	Y	Y	Y	Y
Were confounding factors identified?	U	N	N	N	U	U	N
Were strategies to deal with confounding factors stated?	N	N	N	N	N	N	N
Were the groups/participants free of the outcome at the start of the study (or at the moment of exposure)?	Y	Y	Y	Y	Y	Y	Y
Were the outcomes measured in a valid and reliable way?	Y	Y	Y	Y	Y	Y	Y
Was the follow up time reported and sufficient to be long enough for outcomes to occur?	Y	Y	Y	Y	Y	Y	Y
Was follow up complete, and if not, were the reasons to loss to follow up described and explored?	U	Y	Y	Y	U	Y	Y
Were strategies to address incomplete follow up utilized?	N	N	Y	U	N	N	N
Was appropriate statistical analysis used?	Y	Y	Y	Y	Y	Y	Y
**CASE SERIES**	**Chang** [30]	**He** [32]	**Margabandu** [34]	**Qu** [36]	**Ruhin** [37]	**Shen** [38]	
Were there clear criteria for inclusion in the case series?	Y	Y	Y	Y	Y	Y	
Was the condition measured in a standard, reliable way for all participants included in the case series?	Y	Y	Y	Y	Y	Y	
Were valid methods used for identification of the condition for all participants included in the case series?	Y	Y	Y	Y	Y	Y	
Did the case series have consecutive inclusion of participants?	U	U	Y	N	Y	Y	
Did the case series have complete inclusion of participants?	Y	Y	Y	Y	Y	Y	
Was there clear reporting of the demographics of the participants in the study?	Y	Y	Y	N	Y	Y	
Was there clear reporting of clinical information of the participants?	Y	Y	Y	N	Y	Y	
Were the outcomes or follow up results of cases clearly reported?	Y	Y	Y	Y	Y	Y	
Was there clear reporting of the presenting site(s)/clinic(s) demographic information?	Y	Y	Y	N	Y	Y	
Was statistical analysis appropriate?	Y	NA	Y	Y	NA	U	
**CASE REPORTS**	**Berrone** [26]	**Chang** [28]	**Li** [33]	**Paranque** [35]			
Were patient’s demographic characteristics clearly described?	Y	U	Y	Y			
Was the patient’s history clearly described and presented as a timeline?	N	N	Y	Y			
Was the current clinical condition of the patient on presentation clearly described?	Y	Y	Y	Y			
Were diagnostic tests or assessment methods and the results clearly described?	Y	Y	Y	Y			
Was the intervention(s) or treatment procedure(s) clearly described?	Y	Y	Y	Y			
Was the post-intervention clinical condition clearly described?	Y	Y	Y	Y			
Were adverse events (harms) or unanticipated events identified and described?	Y	N	Y	Y			
Does the case report provide takeaway lessons?	Y	Y	Y	Y			

This table summarizes the risk of bias assessment for the included cohort studies, case series, and case reports evaluated using the JBI appraisal tool. The table categorizes the studies by their type and lists key criteria used to evaluate the risk of bias, including the identification of confounding factors, validity and reliability of measurements, completeness of follow-up, and appropriateness of statistical analysis. Each cell contains a “Y” (yes), “N” (no), “U” (unclear), or “NA” (not applicable), indicating whether the specific criterion was met. The assessment highlights the methodological strengths and potential biases within the studies, aiding in the interpretation of the overall evidence quality in this systematic review.

**Table 3:** Summary of Study Characteristics and Outcomes.

AUTHORS	MALIGNANCIES	%	RADIATION THERAPY	FLAP SURVIVAL	IMPLANTS FAILURE	MANDIBLE DEFECT (AVERAGE)
Antúnez-Conde et al. [25]	2	40	2	5 (100%)	2	84–106 mm (92)
Berrone et al. [26]	0	0	0	1(100%)	0	*
Bianchi et al. [27]	0	0	0	9 (100%)	*	40–50 mm (39)
Chang et al. (1) [28]	0	0	0	1 (100%)	0	*
Chang et al. (2) [29]	1	8	0	13 (100%)	0	*
Chang et al. (3) [30]	0	0	0	10 (100%)	*	50–100 mm (73.5)
Chen et al. [31]	2	17	0	12 (100%)	0	*
Cuéllar et al. [15]	5	63	5	8 (100%)	1	68–102 mm (85.3)
He et al. [32]	3	43	3	7 (100%)	0	65–100 mm (80.7)
Li et al. [33]	1	100	0	1 (100%)	0	*
Margabandu et al. [34]	10	38	10	26 (100%)	0	60–100 mm (83.8)
Paranque et al. [35]	0	0	0	1 (100%)	0	*
Qu et al. [36]	5	9.6	*	50/52 (96%)	4	*
Ruhin et al. [37]	1	33	1	3 (100%)	0	50–100 mm (75)
Shen et al. [38]	5	11	1	44/45 (97.7%)	*	*
Trilles et al. [39]	8	19	3	41/42 (97.6%)	*	*
Wang et al. [40]	0	0	0	9 (100%)	0	*
				**241 (98.3%)**	**7**	**Average: 75.6 mm**

This table presents the characteristics and key outcomes of the included studies. It includes the number and percentage of patients with malignancies, the use of radiation therapy, flap survival rates, implant failure rates, and the average mandibular defect size. The studies are listed by author, with the number of malignancies and radiation therapy sessions reported. Flap survival is shown as the number of successful integrations versus total flaps, and implant failures are noted where applicable. The average mandibular defect size is provided in millimeters for each study. Overall, the table highlights a flap survival rate of 98.3% and an implant failure rate of 1.74%, with the average mandibular defect size being 75.6 mm.* Not specified nor mentioned in the article.

**Table 4:** GRADE certainty assessment for key outcomes.

Certainty Assessment							Impact	Certainty	Importance
№ of Studies	Study Design	Risk of Bias	Inconsistency	Indirectness	Imprecision	Other Considerations			
**FLAP SURVIVAL (assessed with: Specified as necrosis, or the need for a new flap)**									
17	non-randomised studies	not serious	not serious	not serious	not serious	all plausible residual confounding would reduce the demonstrated effect	Considering the observational nature of the studies and the consistent, direct, and precise reporting of flap survival rates, the overall certainty for the outcome of flap survival is moderate. Flap survival was rated at 98.3% with a total flap loss of 4	⨁⨁⨁◯ Moderate	IMPORTANT
**IMPLANT FAILURE (assessed with: Stated)**									
17	non-randomised studies	not serious	not serious	Serious ^a^	not serious	all plausible residual confounding would reduce the demonstrated effect	Considering the observational nature of the studies, the omission of implant failure data in 2 studies, and the potential for publication bias, the overall certainty for the outcome of implant failure is low.	⨁⨁◯◯ Low	IMPORTANT
**ESTHETICS (assessed with: Subjective findings)**									
16	non-randomised studies	serious	Serious ^b^	very serious ^c^	Serious ^d^	all plausible residual confounding would reduce the demonstrated effect	Three studies directly addressing cosmetic results found good to excellent outcomes in 76 patients using a standardized protocol for quantitative assessment. Seven studies mentioned achieving satisfactory facial contour and good aesthetic results qualitatively. Six studies only discussed aesthetic results in their discussions or conclusions. One study did not mention aesthetics at all.	⨁◯◯◯ Very low	IMPORTANT

Explanations: ^a^ Since 2 studies do not address implant failure, the evidence is somewhat indirect for this outcome. ^b^ Facial esthetics and function are not systematically measured. Only 3 groups have devised a grading system for esthetics (Chen et al. [31], Margabandu et al. [34], Shen et al. [38]). 6 articles mention a good esthetic result, without offering further data. 6 articles mention good results in their discussion or conclusions. ^c^ Since not all studies may directly address combined cosmetic and functional outcomes or use standardized measures, the evidence may be somewhat indirect. ^d^ Results were determined in a subjective fashion, and were only standardized in 3 articles, using subjective scales. This table presents the GRADE certainty assessment for the key outcomes of flap survival, implant failure, and esthetics in patients undergoing mandibular reconstruction with a double-barreled fibula flap (DBFF). The table evaluates the number of studies, study design, risk of bias, inconsistency, indirectness, imprecision, and other considerations to determine the overall certainty of evidence for each outcome. Flap Survival: Assessed with criteria such as necrosis or the need for a new flap, involving 17 non-randomized studies. The overall certainty is rated as moderate due to the observational nature of the studies, despite consistent, direct, and precise reporting. Implant Failure: Assessed with explicitly stated failure rates across 17 non-randomized studies. The overall certainty is rated as low due to the omission of implant failure data in 2 studies and potential publication bias. Esthetics: Assessed using subjective findings from 16 non-randomized studies. The overall certainty is rated as very low due to serious risk of bias, inconsistency, very serious indirectness, and imprecision in the subjective assessments.

## Data Availability

The data that support the findings of this study are available from the corresponding author upon reasonable request.

## Appendix A

Following the EMBASE PICO form, the search query yielded the following:

(disease, head and neck’/exp OR ‘disease, head and neck’ OR ‘head and neck disease’/exp OR ‘head and neck disease’ OR ‘head neck disease’/exp OR ‘head neck disease’ OR ‘chin malformation’/exp OR ‘chin malformation’ OR ‘dysostosis mandibularis’/exp OR ‘dysostosis mandibularis’ OR ‘jaw abnormalities’/exp OR ‘jaw abnormalities’ OR ‘jaw anomaly’/exp OR ‘jaw anomaly’ OR ‘jaw deformity’/exp OR ‘jaw deformity’ OR ‘jaw disformity’/exp OR ‘jaw disformity’ OR ‘jaw malformation’/exp OR ‘jaw malformation’ OR ‘malformation, chin’/exp OR ‘malformation, chin’ OR ‘malformation, jaw’/exp OR ‘malformation, jaw’ OR ‘mandible deformity’/exp OR ‘mandible deformity’ OR ‘mandibular condyle agenesis’/exp OR ‘mandibular condyle agenesis’ OR ‘mandibular defect’/exp OR ‘mandibular defect’ OR ‘mandibular dysostosis’/exp OR ‘mandibular dysostosis’ OR ‘mandibular malformation’/exp OR ‘mandibular malformation’) AND (‘fibula autograft’/exp OR ‘fibula autograft’ OR ‘fibula flap’/exp OR ‘fibula flap’ OR ‘fibula flaps’/exp OR ‘fibula flaps’ OR ‘fibula graft’/exp OR ‘fibula graft’ OR ‘fibula grafts’/exp OR ‘fibula grafts’ OR ‘fibula osteocutaneous flap’/exp OR ‘fibula osteocutaneous flap’ OR ‘fibula osteocutaneous flaps’/exp OR ‘fibula osteocutaneous flaps’ OR ‘fibula transplant’/exp OR ‘fibula transplant’ OR ‘fibula transplantation’/exp OR ‘fibula transplantation’ OR ‘fibular bone flap’/exp OR ‘fibular bone flap’ OR ‘fibular bone flaps’/exp OR ‘fibular bone flaps’ OR ‘fibular bone graft’/exp OR ‘fibular bone graft’ OR ‘fibular bone grafts’/exp OR ‘fibular bone grafts’ OR ‘fibular flap’/exp OR ‘fibular flap’ OR ‘fibular flaps’/exp OR ‘fibular flaps’ OR ‘fibular graft’/exp OR ‘fibular graft’ OR ‘fibular grafts’/exp OR ‘fibular grafts’ OR ‘fibular osteocutaneous flap’/exp OR ‘fibular osteocutaneous flap’ OR ‘fibular osteocutaneous flaps’/exp OR ‘fibular osteocutaneous flaps’) AND (‘adult’/exp OR ‘adult’) AND (‘fibula autograft’/exp OR ‘fibula autograft’ OR ‘fibula flap’/exp OR ‘fibula flap’ OR ‘fibula flaps’/exp OR ‘fibula flaps’ OR ‘fibula graft’/exp OR ‘fibula graft’ OR ‘fibula grafts’/exp OR ‘fibula grafts’ OR ‘fibula osteocutaneous flap’/exp OR ‘fibula osteocutaneous flap’ OR ‘fibula osteocutaneous flaps’/exp OR ‘fibula osteocutaneous flaps’ OR ‘fibula transplant’/exp OR ‘fibula transplant’ OR ‘fibula transplantation’/exp OR ‘fibula transplantation’ OR ‘fibular bone flap’/exp OR ‘fibular bone flap’ OR ‘fibular bone flaps’/exp OR ‘fibular bone flaps’ OR ‘fibular bone graft’/exp OR ‘fibular bone graft’ OR ‘fibular bone grafts’/exp OR ‘fibular bone grafts’ OR ‘fibular flap’/exp OR ‘fibular flap’ OR ‘fibular flaps’/exp OR ‘fibular flaps’ OR ‘fibular graft’/exp OR ‘fibular graft’ OR ‘fibular grafts’/exp OR ‘fibular grafts’ OR ‘fibular osteocutaneous flap’/exp OR ‘fibular osteocutaneous flap’ OR ‘fibular osteocutaneous flaps’/exp OR ‘fibular osteocutaneous flaps’ OR ‘double barrel’ OR ‘cancer irradiation therapy’/exp OR ‘cancer irradiation therapy’ OR ‘cancer radiation’/exp OR ‘cancer radiation’ OR ‘cancer radiation therapy’/exp OR ‘cancer radiation therapy’ OR ‘cancer radiation, therapeutic’/exp OR ‘cancer radiation, therapeutic’ OR ‘cancer radiotherapy’/exp OR ‘cancer radiotherapy’ OR ‘radiotherapy, cancer’/exp OR ‘radiotherapy, cancer’ OR ‘tumor irradiation’/exp OR ‘tumor irradiation’ OR ‘tumor radiation’/exp OR ‘tumor radiation’ OR ‘tumor radiotherapy’/exp OR ‘tumor radiotherapy’ OR ‘tumour irradiation’/exp OR ‘tumour irradiation’ OR ‘tumour radiation’/exp OR ‘tumour radiation’ OR ‘tumour radiotherapy’/exp OR ‘tumour radiotherapy’) AND (‘bicon’/exp OR ‘bicon’ OR ‘grafton’/exp OR ‘grafton’ OR ‘straumann mini’/exp OR ‘straumann mini’ OR ‘straumann pure’/exp OR ‘straumann pure’ OR ‘swish active’/exp OR ‘swish active’ OR ‘swish tapered’/exp OR ‘swish tapered’ OR ‘variobase’/exp OR ‘variobase’ OR ‘dental implant’/exp OR ‘dental implant’ OR ‘dental implants’/exp OR ‘dental implants’ OR ‘implant, teeth’/exp OR ‘implant, teeth’ OR ‘implant, tooth’/exp OR ‘implant, tooth’ OR ‘implants, teeth’/exp OR ‘implants, teeth’ OR ‘implants, tooth’/exp OR ‘implants, tooth’ OR ‘intramucosal dental implant’/exp OR ‘intramucosal dental implant’ OR ‘teeth implant’/exp OR ‘teeth implant’ OR ‘teeth implants’/exp OR ‘teeth implants’ OR ‘tooth implant’/exp OR ‘tooth implant’ OR ‘tooth implants’/exp OR ‘tooth implants’ OR ‘3m espe lava plus’/exp OR ‘3m espe lava plus’ OR ‘3m lava esthetic’/exp OR ‘3m lava esthetic’ OR ‘advent (tooth prosthesis)’/exp OR ‘advent (tooth prosthesis)’ OR ‘bonartic ct’/exp OR ‘bonartic ct’ OR ‘cerasmart’/exp OR ‘cerasmart’ OR ‘certain bellatek (tooth prosthesis)’/exp OR ‘certain bellatek (tooth prosthesis)’ OR ‘certain ginighue (tooth prosthesis)’/exp OR ‘certain ginighue (tooth prosthesis)’ OR ‘certain iol (tooth prosthesis)’/exp OR ‘certain iol (tooth prosthesis)’ OR ‘certain locator (tooth prosthesis)’/exp OR ‘certain locator (tooth prosthesis)’ OR ‘elliptic matrix’/exp OR ‘elliptic matrix’ OR ‘gingihue’/exp OR ‘gingihue’ OR ‘hex-lock’/exp OR ‘hex-lock’ OR ‘locator f-tx’/exp OR ‘locator f-tx’ OR ‘locator r-tx’/exp OR ‘locator r-tx’ OR ‘mazic duro’/exp OR ‘mazic duro’ OR ‘omniloc’/exp OR ‘omniloc’ OR ‘sphero flex’/exp OR ‘sphero flex’ OR ‘artificial teeth’/exp OR ‘artificial teeth’ OR ‘artificial tooth’/exp OR ‘artificial tooth’ OR ‘ceramic artificial teeth’/exp OR ‘ceramic artificial teeth’ OR ‘dental implant prosthetic teeth bar’/exp OR ‘dental implant prosthetic teeth bar’ OR ‘dental implant suprastructure, permanent, preformed’/exp OR ‘dental implant suprastructure, permanent, preformed’ OR ‘dental prostheses’/exp OR ‘dental prostheses’ OR ‘dental prosthesis’/exp OR ‘dental prosthesis’ OR ‘dental prosthesis retention’/exp OR ‘dental prosthesis retention’ OR ‘dental prosthesis, device (physical object)’/exp OR ‘dental prosthesis, device (physical object)’ OR ‘denture prosthesis’/exp OR ‘denture prosthesis’ OR ‘gold artificial teeth’/exp OR ‘gold artificial teeth’ OR ‘preformed permanent dental implant suprastructure’/exp OR ‘preformed permanent dental implant suprastructure’ OR ‘prostheses, dental’/exp OR ‘prostheses, dental’ OR ‘prosthesis, dental’/exp OR ‘prosthesis, dental’ OR ‘prosthesis, tooth’/exp OR ‘prosthesis, tooth’ OR ‘tooth prosthesis’/exp OR ‘tooth prosthesis’ OR ‘tooth, artificial’/exp OR ‘tooth, artificial’ OR ‘zerion gi’/exp OR ‘zerion gi’ OR ‘zerion html’/exp OR ‘zerion html’ OR ‘zerion utml’/exp OR ‘zerion utml’ OR ‘blade implantation’/exp OR ‘blade implantation’ OR ‘dental implantation’/exp OR ‘dental implantation’ OR ‘dental implantation, endosseous’/exp OR ‘dental implantation, endosseous’ OR ‘dental implantation, endosseous, endodontic’/exp OR ‘dental implantation, endosseous, endodontic’ OR ‘dental implantation, subperiosteal’/exp OR ‘dental implantation, subperiosteal’ OR ‘endodontic endosseous dental implantation’/exp OR ‘endodontic endosseous dental implantation’ OR ‘endosseous dental implantation’/exp OR ‘endosseous dental implantation’ OR ‘immediate dental implant loading’/exp OR ‘immediate dental implant loading’ OR ‘subperiosteal dental implantation’/exp OR ‘subperiosteal dental implantation’ OR ‘tooth implantation’/exp OR ‘tooth implantation’) AND (‘care, dental’/exp OR ‘care, dental’ OR ‘care, tooth’/exp OR ‘care, tooth’ OR ‘community dentistry’/exp OR ‘community dentistry’ OR ‘comprehensive dental care’/exp OR ‘comprehensive dental care’ OR ‘dental care’/exp OR ‘dental care’ OR ‘dental care for aged’/exp OR ‘dental care for aged’ OR ‘dental care for children’/exp OR ‘dental care for children’ OR ‘dental care for chronically ill’/exp OR ‘dental care for chronically ill’ OR ‘dental care for disabled’/exp OR ‘dental care for disabled’ OR ‘dental care for handicapped’/exp OR ‘dental care for handicapped’ OR ‘dental care program’/exp OR ‘dental care program’ OR ‘dental care programme’/exp OR ‘dental care programme’ OR ‘dental caries activity tests’/exp OR ‘dental caries activity tests’ OR ‘dental esthetics’/exp OR ‘dental esthetics’ OR ‘dental health care’/exp OR ‘dental health care’ OR ‘dental health services’/exp OR ‘dental health services’ OR ‘dental high-speed technique’/exp OR ‘dental high-speed technique’ OR ‘dental procedure’/exp OR ‘dental procedure’ OR ‘dental service’/exp OR ‘dental service’ OR ‘dental service, hospital’/exp OR ‘dental service, hospital’ OR ‘dental stress analysis’/exp OR ‘dental stress analysis’ OR ‘dental technique’/exp OR ‘dental technique’ OR ‘dental treatment’/exp OR ‘dental treatment’ OR ‘denture identification marking’/exp OR ‘denture identification marking’ OR ‘electrogalvanism, intraoral’/exp OR ‘electrogalvanism, intraoral’ OR ‘enamel microabrasion’/exp OR ‘enamel microabrasion’ OR ‘esthetics, dental’/exp OR ‘esthetics, dental’ OR ‘hospital dental service’/exp OR ‘hospital dental service’ OR ‘intraoral electrogalvanism’/exp OR ‘intraoral electrogalvanism’ OR ‘tooth bleaching’/exp OR ‘tooth bleaching’ OR ‘tooth care’/exp OR ‘tooth care’ OR ‘tooth remineralization’/exp OR ‘tooth remineralization’ OR ‘osseo-integration’/exp OR ‘osseo-integration’ OR ‘osseointegration’/exp OR ‘osseointegration’ OR ‘osseous integration’/exp OR ‘osseous integration’ OR ‘osseointegrated implant’/exp OR ‘osseointegrated implant’ OR ‘beauty’/exp OR ‘beauty’ OR ‘esthetic value’/exp OR ‘esthetic value’ OR ‘esthetics’/exp OR ‘esthetics’ OR ‘implant survival’/exp OR ‘implant survival’ OR ‘patient satisfaction’/exp OR ‘patient satisfaction’).
